# Comparison of Bone-Patella Tendon-Bone and Four-Strand Hamstring Tendon Grafts for Anterior Cruciate Ligament Reconstruction: A Prospective Study

**DOI:** 10.7759/cureus.19197

**Published:** 2021-11-02

**Authors:** Christina Arida, Chrisovalantis G Tsikrikas, Dimitrios S Mastrokalos, Andreas Panagopoulos, John Vlamis, Ioannis K Triantafyllopoulos

**Affiliations:** 1 Orthopaedic Department, KAT Hospital, National and Kapodistrian University of Athens School of Medicine, Athens, GRC; 2 Department of Knee Rehabilitation, Bioanataxi Physical Therapy Center, Athens, GRC; 3 1st Orthopaedic Department, Attikon Hospital, National and Kapodistrian University of Athens School of Medicine, Athens, GRC; 4 Orthopaedic Department, University of Patras School of Medicine, Patras, GRC; 5 3rd Orthopaedic Department, KAT Hospital, National and Kapodistrian University of Athens School of Medicine, Athens, GRC; 6 5th Orthopaedic Department, HYGEIA Hospital, Athens, GRC

**Keywords:** re-rupture, knee injuries, bone-patella tendon-bone graft, four strand hamstring tendon graft, anterior cruciate ligament (acl) reconstruction

## Abstract

Introduction

To date, the proper choice of graft for anterior cruciate ligament (ACL) reconstruction remains a matter of conflict. We aimed to compare the clinical and functional outcomes of the two most commonly utilized autografts, bone-patella tendon-bone (BPTB) and four-strand hamstring tendon (HT) graft, at 6 and 12 months after surgery.

Methods

In a prospective randomized study, we included a total of 60 patients undergoing ACL reconstruction, thirty in BPTB and thirty in HT group. All patients were amateur athletes and were evaluated at 6 and12 months after surgery for: (a)postoperativefunctionality of the operated knee by the Tegner, the Lysholm and the International Knee Documentation Committee (IKDC) scoring scales, (b) anterior cruciate ligament (ACL) instability of the operated knee compared to the healthy contralateral knee by the KT-1000 arthrometer and (c) theextension and flexion muscle strength of the operated knee by a CYBEXisokinetic dynamometer.* *

Results

Patients in the two groups did not differ regarding demographics, and pre-injury functionality status. Significantly more patients in the HT group (n=6) compared to the BPTB group (n=1) experienced ACL re-rupture and underwent revision surgery before follow-up end (p=0.044). All patients, regardless of graft, showed significant improvement within each group of functional assessments by Lysholm, Tegner and IKDC scores, as well as of Cybex measurements -with an increase of peak torque at 60° extension and 180°extension and 60° flexion and 180° flexion- at 12 months compared to 6 months follow-up (p<0.05). However, there was no difference between the two groups regarding knee function improvement or extension measurements neither at 6 nor 12 months. Contrarily, the BPTB graft group had higher values of peak torque (Nm) at 60° and 180° flexion compared to the HT group, both at 6 (p=0.014 and 0.029, respectively) and 12 months (p=0.033 and 0.030, respectively). Postoperative stability was similar between the two groups at 12 months (p=0.519).

Conclusion

Both BPTB and HT grafts present with benefits and drawbacks and remain viable autograft options for primary ACL reconstruction as each has, although HT grafts seem to be more susceptible to re-rupture. The graft selection should be based on the needs and activities of each patient.

## Introduction

Anterior cruciate ligament (ACL) injury is one of the most common injuries of the knee and is responsible for approximately 50% of the total knee reconstruction cases [1]. ACL injury often results in knee joint laxity, altered movement, reduced functionality and a various degree of feeling of pain so the patient has many limitations in daily or sports activities and thus ACL reconstruction is mandatory [2,3].

Graft choice for anterior cruciate ligament reconstruction is crucial, since it is one of the main factors for a successful outcome, but the optimal graft source remains a topic of controversy. The primary goal of surgery is to achieve a functionally stable knee while minimizing morbidity and complications associated with the procedure. The autograft of bone-patella tendon-bone (BPTB) and the autograft of four-strand hamstring tendon, are the most commonly used ones on ACL reconstruction around the world [4-6].

Historically, the BPTB autograft was the gold standard for ACLR, as it allowed proper bone-to-bone tunnel healing, involved a short fixation distance, and provided excellent biomechanical strength [7]. Studies have shown that BPTB autograph had a higher incidence of return to sports activity and a lower rate of revision [7,8] despite the well-documented morbidities including anterior knee pain, difficulty in kneeling, possible patellar fracture and patellar tendon rupture and extension loss [8-10]. The main complication related to BPTB harvesting is anterior knee pain, reported in up to 46% of cases and the surgical violation of the extensor mechanism during graft harvest is the most likely explanation for this difference [8-11].

The hamstrings tendon (HT) autograft has been developed as an alternative to BPTB autograft, resulting in no implications from the extensor apparatus, and less anterior knee pain [8,9,11,12]. A study by Mastrokalos et al. [13] showed that a high rate of patients had pain, loss of sensitivity, or both at the donor site after ACL reconstruction with a BPTB graft, with most experiencing these symptoms up to almost two or three years after the operation. However, studies have shown that it could lead to a decrease in knee stability and flexor weakness compared to BPTB and also to increased risk of infection [9-11,14,15].

Given the significance of the appropriate autograft, studies have proposed algorithms for graft selection. The use of BPTB is recommended for the young high school and college athletes, professional athletes and generally those who have high requirements of activity level and who have no contraindication (eg, patella baha, very thin patellar tendon, significant patellar tendinosis), whereas the use of a hamstring autograft for less demanding younger athletes, older patients or those who have strict requirements for kneeling and knee stretching [8,9].

The purpose of this study is to evaluate the clinical results and describe the outcome of a one-year follow-up of ACL reconstruction between the two most commonly used autografts, the BPTB graft and the hamstring tendon graft in terms of knee laxity, graft failure, flexion and extension torque of the knee and the functionality of the joint in patient’s daily and sports activities. The hypothesis was that BPTB autograft may provide a slightly superior outcome regarding stability and resilience compared to HT autograft, due to its firmer bone-to-bone fixation and its behavior as a strong anelastic ligament, therefore re-rupture incidence would be lower and stability tests would be superior in patients using BPTB autografts.

## Materials and methods

This prospective, comparative and randomized study involved 60 consecutive patients, who were clinically and radiologically diagnosed with ACL rapture. All patients were amateur athletes, with a Tegner scaling score of at least six (≥6), that sustained ACL rupture during sport activities. Patients were excluded if they had bilateral ACL injuries, multi-ligament injuries, articular cartilage lesions greater than ICRS-II, partial meniscectomy more than 25% of total meniscus, meniscal suture repair or previous injuries/surgeries on the affected knee. Patients were prospectively randomized to receive either a BPTB autograft or an HT autograft. Randomization was done by the date of the surgery: on odd number date patients would receive BPTB autograft and on even number date an HT autograft.

All patients were assessed at 6 and 12 months after surgery for: (a) postoperative functional outcomes using the Tegner, Lysholm and International Knee Documentation Committee (IKDC) scoring scales, (b) postoperative knee laxity compared to the normal contralateral one using the KT-1000 arthrometer and (c) muscle function of the operated compared to the normal contralateral one using the Cybex dynamometer. Both KT-1000 and Cybex dynamometer tests were performed by the same physical therapist (V.T.).

For the KT-1000 arthrometer (Medmetric company) the patient was placed in the supine position on an examination table. A bolster (provided with the KT1000) was placed under the thighs so that the knees remained at approximately 25° of flexion. While placing the patient, the heels were positioned symmetrically on a positioning cup (also provided with the KT1000) which places the tibia at 15° external rotation. Once the correct positioning was achieved, the examiner placed the device on the knee of the patient and knee joint laxity was recorded at the manual maximum force.

The Cybex 6000 human version 2004 isokinetic dynamometer was used to evaluate average peak torque at the angular velocities of 60° and 180° of the operated knee. Each test began with a warm-up period of 15 min walking on a treadmill at a normal phase of approximately 5 km/h. While seated in the Cybex dynamometer, subjects performed five sub-maximal extensions and flexions for warming up. Each test consisted of a trial phase of three continuous concentric-eccentric cycles, one-minute rest, and an actual test phase of five continuous concentric-eccentric cycles. The first two trial cycles were of submaximal force, and the final one was at maximal force. Subjects received standardized verbal encouragement to produce maximal efforts throughout the five cycles of the actual test phase. The knee extensor muscle group of the operated limb was tested first at 60° and then at 180° with a 2 min rest between the two speeds. Each subject was instructed to extend the knee from 90° to 5° of flexion against the tibia pad of the dynamometer arm during the concentric phase, and then to resist the dynamometer as it pushed in the opposite direction from 5° to 90° of knee flexion during the eccentric phase. Each subject was instructed to flex the knee from 5° to 90° during the concentric phase, and then to resist the dynamometer from 90° to 5° of knee flexion during the eccentric phase.

All patients gave their informed consent prior to their inclusion in the study. The study was approved by the Ethical Committee of the Medical School, National and Kapodistrian University of Athens, Greece (protocol number: 114, 21/05/2019).

Surgical technique

The same surgeon performed arthroscopic ACL reconstruction to all patients using the same technique.

ACL Reconstruction With BPBT Graft

Under general anesthesia and additional local anesthetic infiltration, use of tourniquet and antibiotic administration, graft harvest was performed through a midline knee incision. Then the remaining patellar tendon and its paratenon were closed with undyed no 2-0 absorbable sutures with side-to-side repair. Two arthroscopic portals (AL and AM) were made under the open skin incision and through the joint capsule. After a global inspection and evaluation of the joint, reconstruction of the torn ACL was performed with an outside-in tibial tunnel and an inside-out femoral tunnel performed either transtibial or through a medial portal. The graft was fixed with interference screws, metal or absorbable. The BPTB bony pegs and the tunnels’ length were kept up to 25 mm. The femoral peg diameter was 9mm and the tibial one was 10 mm. 

ACL Reconstruction with Hamstrings Graft

Under general anesthesia and additional local anesthetic infiltration, use of tourniquet and antibiotic administration, graft harvest was performed through an oblique incision over the pes anserinus. Both semitendinosus and gracilis tendons were harvested and a quadruple graft was prepared with a diameter above 7 mm. Then, two arthroscopic portals (AL and AM) were made and a global inspection and evaluation of the joint was initially performed. Reconstruction of the torn ACL was achieved by the creation of an outside-in tibial tunnel and an inside-out femoral tunnel made through the medial portal. The graft was then inserted and fixed with an adjustable or fixed loop extracortical button suspensory mechanism at the femoral site and an absorbable interference screw at the tibial site. In some cases, a secondary tibial fixation was performed with the use of a cortical buckle.

Rehabilitation protocol

All patients followed the same rehabilitation protocol as shown in Table 1.

**Table 1:** Rehabilitation protocol. CPM: continuous passive motion.

Timeline	Guidelines	Target/aim
Phase 1: 1st postoperative day- departure from hospital	CPM to tolerable angles, Ice therapy, Mobilization of the patella, Exercises for the muscles of the ankle, Passive knee flexion at the edge of the bed, Static contractions of the quadriceps and hamstrings, Lift of a stretched limb in prone position, Partial loading of the limb with crutches, Heel raises exercises	Reduction of inflammation, Try to gain full extension, Good blood circulation, Early mobilization
Phase 2: departure from hospital-10th postoperative day	Static bike without or with minimal resistance, Gradual increase of loading of the limb, Learning to walk gradually without crutches, Climbing low stairs, Active extension and flexion, Exercise of other muscle groups	Increase range of motion, Gradual increase of the load on the limb, Improving muscle strength and endurance
Phase 3: 10th day-6th week	Full load on the limb, Walking in multiple directions, Stairs: ascent, descent, Stretching of quadriceps and hamstrings, Leg press with repetitions, Early plyometric exercises, Proprioception, Exercise all the muscles of the leg, Exercise of the upper body	Progressive return to activities, Prevention of tissue growth and articular fibrosis, Prevention of stiffness, Restoration of normal gait Improvement of muscle strength and endurance, Improvement of proprioception, Maintaining good cardiovascular function, Encourage the patient to become independent of aids
Phase 4: 7th week-12th week	Trampette jogging, Intense walking with duration and uphill/ downhill, Isokinetic exercises of quadriceps and hamstrings	Increase muscle strength and full range of motion, Improvement of isometric power of quadriceps and hamstrings
Phase 5: 13th week-5th month	Exercises of quadriceps and hamstrings with repetitions and resistance, Plyometric exercises, Bounces with change of direction, Jogging, Running Progressive: speed changes, pivoting	Return to specific sports and activities
Phase 6: 6th month	Training and participation in sports without contact with an opponent	Physical and psychological preparation for returning to any activity
Phase 7: 7th month	Return to sports with opponent contact	Unlimited activity

Statistical analysis

Data were expressed as mean±standard deviation (SD) for continuous variables and as frequencies (n), percentages (%) for categorical variables. The Kolmogorov-Smirnov test was utilized for normality analysis of the parameters. Comparisons between the two different grafts at 6 and 12 months respectively were made by using the Student t-test or Mann-Whitney in case of violation of normality. Paired samples t-test or Wilcoxon test, in case of violation of normality, were used for the comparison of different time measurements (6 vs. 12 months) of variables for each graft separately. All tests are two-sided, statistical significance was set at p < 0,05. All analyses were carried out using the statistical package SPSS ver 21.00 (IBM Corporation, Somers, NY, USA).

## Results

Following randomization, 30 of the patients (aged 29.83±11.11, 77% men) underwent a BPTB reconstruction while the other 30 (30.03±11.70, 60% men) underwent hamstring ACL reconstruction. Patients’ demographics, as well as pre-injury functionality status measurements, were similar between the two groups (Table 2).

**Table 2:** Demographics and pre-injury functional scores of 30 patients using BPTB grafts and 30 patients using HT grafts for ACL reconstruction. IKDC: International Knee Documentation Committee; BPTB: bone-patella tendon-bone; ACL: anterior cruciate ligament.

	Total (n = 60)	BPTB (n = 30)	Hamstrings (n = 30)	p-value
Age	29.93±11.31	29.83±11.11	30.03±11.70	0.946
Sex woman/man, n	19/41	7/23	12/18	0.267
Operated Knee right/left, n	32/28	16/14	16/14	1.000
IKDC pre-injury	100±0	100±0	100±0	1.000
Lysholm pre-injury	99.9±0.73	99.9±0.73	99.9±0.73	1.000
Tegner pre-injury	8.27±1.11	8.43±.1.04	8.10±1.18	0.580

Outcome data, including functional score and objective measurements, were generated for all 60 patients at 6 months, and for 53 of them at 12 months. The remaining seven patients - one in the BPTP group and six in the HTs group (p=0.044) - experienced ACL re-rupture in the operated knee and underwent revision surgery before follow-up ends.

**Figure 1:**
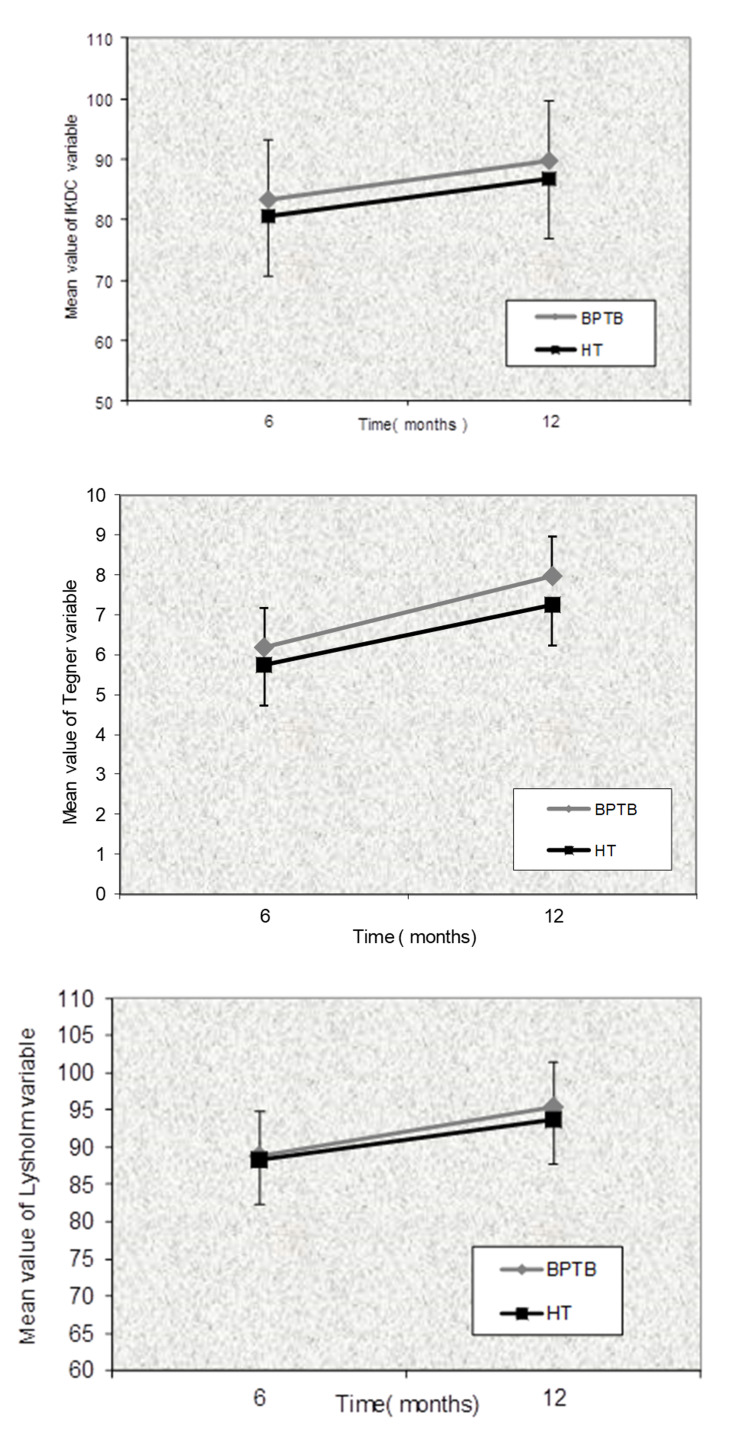
Progress of the values of IKDC, Tegner and Lysholm functional scores from 6 to 12 months for all patients with BPTB and HT grafts. IKDC: International Knee Documentation Committee; BPTB: bone-patella tendon-bone; HT: hamstring tendon.

Both groups demonstrated significant improvement at 12 months assessment compared to 6 months assessment with respect to Lysholm, Tegner and IKDC scores. However, we found no statistically significant differences between the two groups as regards to knee function improvement neither at 6 months nor 12 months (Figure 1 & Table 3). More specifically there is no statistically significant difference between the 2 grafts for the evaluation of 6 months (p=0.183) and 12 months (p=0.088) for Tegner variable, for the evaluation of 6 months (p=0.367) and 12 months (p=0.350) for the IKDC variable and for the evaluation of 6 months (p=0.771 ) and 12 months (p=0.284) for the variable Lysholm but there was a statistical increase from 6 to 12 months of all three variables for the BPTB group (p < 0.005) and the HTs group (p<0.005) taking into account the Bonferroni correction.

**Table 3:** Function measurements using Tegner, IKDC and Lysholm scaling scores and Cybex scores for extension and flexion at 60˚ and 180˚ measured at 6 and 12 months in all patients with BPTB and HT grafts. IKDC: International Knee Documentation Committee; BPTB: bone-patella tendon-bone; HT: hamstring tendon.

			6 months	12 months	p-value_within group_
Tegner		BPTB (n=29)	6.14±1.33	7.97±1.43	<0.005
		Hamstrings (n=24)	5.64±1.25	7.24±1.64	<0.005
IKDC		BPTB (n=29)	83.28±10.45	89.71±10.48	<0.005
		Hamstrings (n=24)	79.64±12.26	86.88±11.59	<0.005
Lysholm		BPTB (n=29)	88.79±9.31	95.34±6.31	<0.005
		Hamstrings (n=24)	87.64±6.73	93.68±4.71	<0.005
Cybex scores	PT Extension 60^0^	BPTB (n=29)	125.59±58	153.17±50.84	<0.005
		Hamstrings (n=24)	123.38±43.49	138.08±48.91	<0.005
	PT Flexion 60^0^	BPTB (n=29)	98.34±32.07	104.28±31.72	0.055
		Hamstrings (n=24)	79.29±23.07	86.17±27.84	0.016
	PT Extension 180^0^	BPTB (n=29)	93.07±38.40	109.90±33.52	<0.005
		Hamstrings (n=24)	85.04±30.56	99.79±30.32	<0.005
	PT Flexion 180^0^	BPTB (n=29)	77.86±26.37	85.83±22.36	0.017
		Hamstrings (n=24)	63.13±20.95	71.38±24.62	0.001

Regarding postoperative stability, measured using the KT-1000 arthrometer at 12 months, we found no statistically significant difference between the two groups (p=0.519).

**Figure 2:**
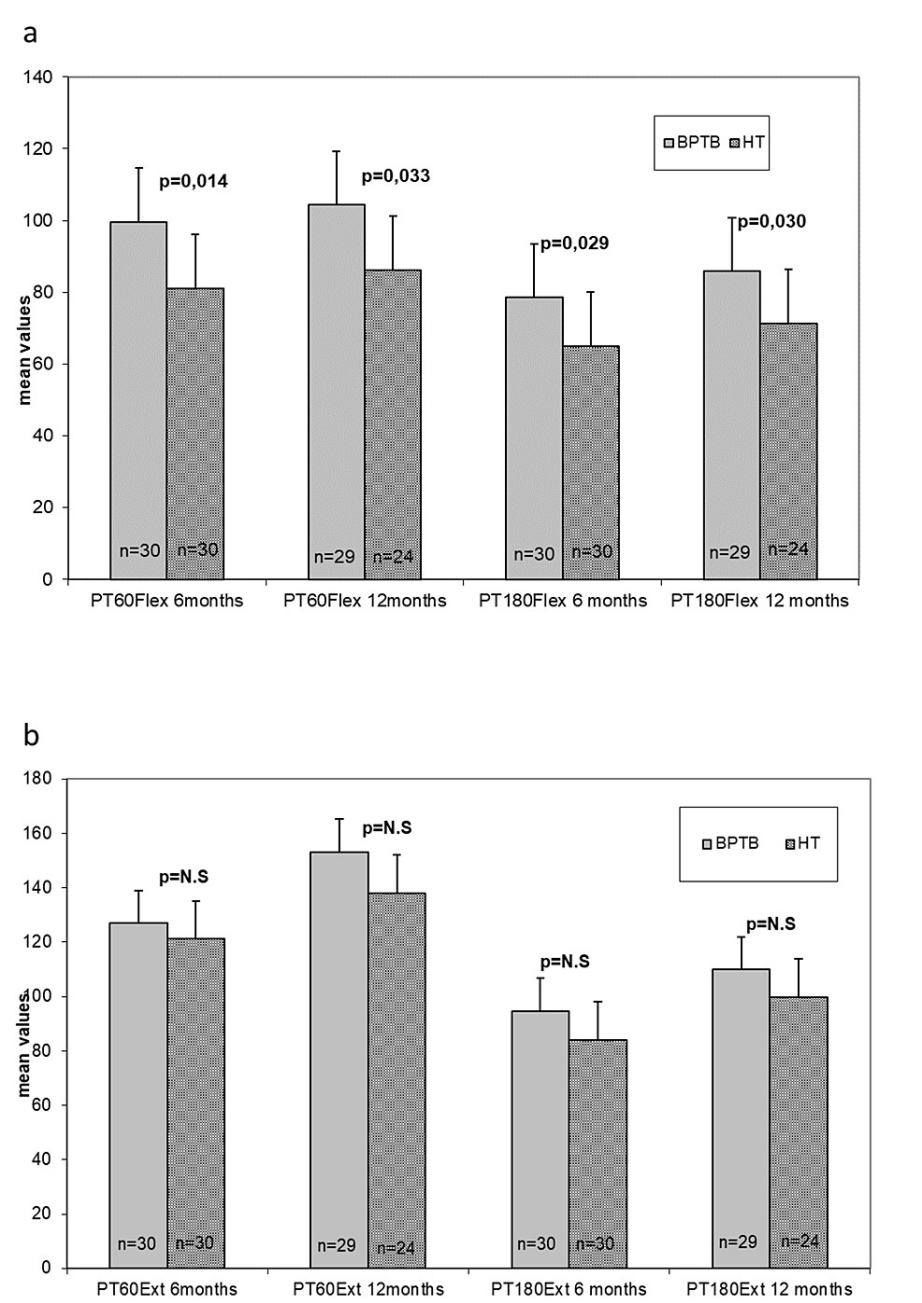
Peak torque values of flexion (a) and extension (b) at 60˚ and 180˚ measured at 6 and 12 months in all patients with BPTB and HT grafts. BPTB: bone-patella tendon-bone; HT: hamstring tendon.

Finally, measurements with the Cybex isokinetic dynamometer were similar between the BPTB and HT graft groups at 6 and 12 months, except for flexion measurements at both 60° and 180°, which were better for the BPTB group. The BPTB had higher values of peak torque (Nm) at 60° flexion at 6 months (p=0.014) and at 12 months (p=0.033), value of peak torque (Nm) at 180° flexion at 6 months (p=0.029) and at 12 months (p=0.030) compared to the Hamstrings group but there was no difference for value of peak torque (Nm) at 60° extension and for the value of peak torque (Nm) at 180° extension between the two groups at 6 and 12 months (Figure 2a and 2b).

Cybex also showed that all patients, regardless of graft, had significantly improved measurements at 12 months assessment compared to their 6 months measurements. More specifically we noted that there was a statistically significant increase from 6 months to 12 months for value of peak torque (Nm) at 60° extension and 180°extension, and for value of peak torque (Nm) at 60° flexion and 180° flexion at the BPTB group (p<0.05) and at the Hamstrings group (p<0.05) for the operated knee (Figure 2 & Table 3).

## Discussion

This study set out to compare the subjective functional results, clinical outcomes and objective physical examination findings in a population of amateur athletes who had undergone ACL reconstruction with the more commonly used autografts of BPTB and HTs.

Regarding patient-reported outcomes, as reflected by the Tegner, Lysholm and IKDC scaling scores, we found no significant differences between the two groups. Our findings are in line with findings from previous studies, that also reported similar functional knee scores between the two grafts [9,16-21]. Two prospective studies, with a 10-year and 20-year follow-up respectively, reported similar clinical outcomes between the two grafts evaluated by the IKDC score [17,18] Similarly, an older review reported no statistically significant differences between the two graft choices regarding neither IKDC, Tegner nor Lysholm scores [12]. On the contrary, in a recent randomized clinical trial of 5-year follow-up, Mohtadi et al. [20] showed that, while there was no difference among the groups at five years considering Tegner scores, there was a trend towards a higher percentage of normal and nearly normal IKDC grades in the patellar tendon group compared with the hamstring tendon. To the best of our knowledge, there is no study reporting on Lysholm scores that has shown significant graft differences after BPBT versus HT reconstructions.

Numerous studies have focused on knee stability after ACL reconstruction, and the results are quite contradictory. In accordance with our findings, older and more recent meta-analyses have also reported similar instrumented laxity between BPBT and HT reconstructions [9,18,21,22] in contrast to the Cochrane review by Mohtadi et al. [12] at 2011 and the most recent review by Schuette et all. [15] at 2017 in which they demonstrated that a number of previous trials favored BPBT graft for instrumented laxity testing. A statistical difference of postoperative KT-1000 in favor of BPTB autografts was also found in a metanalysis made by Li et al. [10] while a metanalysis by Xie et al. [8] showed that there was an apparent but non-significant difference in postoperative KT-1000/2000 between BPTB and HT autografts in the reconstruction of ACL (p = 0.06). To the best of our knowledge, there is only one meta-analysis by Prodromos et al. [5] that showed higher ACLR stability rates with HT grafts than with BPTB grafts, but it is important to specify that it was the first to separate out obsolete 2-strand HT grafts from the currently used 4-strand HT grafts and show that 4HT grafts produce higher stability rates than 2HT.

In a review by Vaishya et al. BPTB graft was associated with better postoperative knee stability and a higher rate of returning to high-level sports but also higher rates of morbidity [4].

An important finding of our study with respect to graft survival, was that we found significant more failures in the HT group (20%) as compared with the BPTB group (3,3%) (p=0.044). This is in accordance with previous studies that found higher rates of failure for patients undergoing ACLR with an HT autograft, even if in some cases this did not reach statistical significance [8-10,12,17,18,23]. On the other hand, Gabler et al. [23] concluded that both BPTB and HT autografts demonstrated a low risk of graft failure and a moderately high rate of return to preinjury activity levels.

Moreover, we found that patients undergoing BPBT graft reconstruction had better measurements to flexion peak torque compared to HT patients and that all patients -in either group- had better results at 12 months compared to 6 months regarding all the evaluation tools used. The first outcome may be attributed to the fact that the gracilis and semitendinosus muscles mainly function as internal tibial rotators and knee flexors, so that HT autograft harvests may lead to flexion strength deficits. In the review by Mohtadi et al. [12] there were many reports of greater flexion strength in the BPTB group but also of the loss of extension strength compared to the HT group, something that didn’t occur in our study. This was also found in a study by Huber et al. [24], where knee extensor strength was lower in patients operated with the BPTB graft at the five-month but not at the nine-month follow-up. In addition, they found that knee flexor strength was lower in patients operated with the HT graft at both their postoperative time evaluation points. Aune et al. [25] showed that while the HT group had better isokinetic knee extension strength and endurance after six months compared with the BPTB group, after 12 and 24 months, no differences were found between the groups. There is actually more consensus in the literature about longer-term strength recovery, as no knee extensor strength deficits were consistently observed in both BPTB and HT patients 6 to 24 months after ACLR [16,24]. Ageberg et al. [26] showed persistent knee flexor strength deficits even three to five years after ACL surgery with the use of HT. In a randomized trial by Webster et al. [27] differences that were apparent between the two grafts at 3-years were no longer apparent at 15 years of evaluation. On the other hand, Leys et al. [28] reported that there was a significantly greater extension deficit rate in the BPTB group at 15-year follow-up, but little differences between HT and BPTB grafts for a variety of clinical outcome and patient-reported variables. Differences in donor site morbidity that were identified at earlier follow-up were not present at 15 years, and patients in the BPTB group tended to participate more frequently in sports [27]. A systematic review by Xergia et al. [29] showed that isokinetic muscle strength deficits, when existed, following ACL reconstruction are associated with the location of the donor site and these deficits appear to be unresolved up to 2 years after ACL reconstruction. Aglietti et al. [16] reported no strength differences between the two groups at 24 months of evaluation. To equalize these differences in muscle strength between graft types specific rehabilitation protocol most suitable in its case is needed.

Regarding patients’ improvement between 6-month and 12-month evaluation, Laxdal et al. [21] also showed a significant improvement between two- and three-year follow-up in both groups in terms of the Lysholm score and Tegner activity level, but again no significant differences between the two groups, indicating that both reconstructions produce similar and, in overall terms, satisfactory knee function, including a significant increase in activity level. In contrast, Aune et al. [25] showed a trend towards better subjective results after six months when an HT graft had been used rather than a BPTP graft; however, these parameters equalized with time.

We should note the significance of the proper timing for the athlete to return to his previous athletic activities. Early return to the pre-injury sports activity can lead to rupture, which was the case in seven of our patients. All the ruptures in our study occurred between 6 and 12 months. Every patient must be evaluated frequently and return gradually to his previous activities. A recent cross-sectional study demonstrated a low rate of returning to sports after ACL reconstruction, with psychological aspects playing a significant role and fear of reinjury being the most frequent cause of not returning to sport [6]. A systematic review and meta-analysis by Ardern et al. [30] showed that on average, 81% of people returned to any sport, 65% returned to their pre-injury level of sport and 55% returned to the competitive level sport after surgery. More specifically playing elite sport and having a positive psychological response favoured returning to the pre-injury level sport. Receiving an HT autograft favoured returning to competitive level sport, whereas receiving a BPTB autograft favoured returning to the preinjury level of sport activities.

Since today, it is not clear which graft of the two is superior, so we have to take a number of factors into consideration in order to choose the most suitable graft for each patient. The best outcome will come after discussion with each patient to evaluate and examine his needs, expectations and participation to specific sports, since some patients may want to avoid the possibility of a knee flexor (e.g., short distance runners) or extensor deficit (e.g., jumpers). Since evidence supports that patients having a BPTB reconstruction are more likely to experience problems in the anterior aspect of their knees, particularly problems with kneeling, BPTB may not be a suitable graft choice for people that kneel a lot such as plumbers or catchers at baseball. Furthermore, the difference in retear rates dictates that HT graft might not be the most suitable choice for high-risk patients, i.e., patients who are young and play cutting and pivoting sports.

This study had some limitations. Although all the patients followed the same rehabilitation protocol after surgery, the quality and consistency of it may have varied without strict and daily supervision and while all patients underwent radiological imaging before surgery, incomplete follow-up radiographic data may limit our ability to draw significant conclusions. Moreover, the follow-up period is relatively small; however, we fell that it is an adequate period of time to safely evaluate functionality and stability outcomes. Finally, although randomisation was dependent on operating days, the operating team and operating procedures did not differ. 

## Conclusions

BPTB and HT grafts used for ACL reconstruction had similar outcomes concerning knee function improvement and Cybex extension measurements, at 6 and 12 months. However, BPTB was found to be superior to the HT as regards to peak torque at 60° and 180° flexion at both 6 and 12 months. Postoperative stability was also similar between the two groups at 12 months follow-up. Within each group, improvement in both functional assessments by Lysholm, Tegner and IKDC scores, as well as in Cybex measurements -with an increase of peak torque at 60˚ extension and 180° extension and 60° flexion and 180° flexion- was observed between 6 to 12 months. Interestingly, graft re-rupture was significantly more common in the HT group than the BPTB group and occurred between 6 to 12 months postoperatively. Conclusively, both graft types offer good knee function and stability and remain viable options for primary ACL reconstruction. Graft selection should be based on the needs and activities of each patient, taking each methods advantages and risk - especially re-rupture probability - into consideration. 

## References

[1] Mall NA, Chalmers PN, Moric M, Tanaka MJ, Cole BJ, Bach BR Jr, Paletta GA Jr (2014). Incidence and trends of anterior cruciate ligament reconstruction in the United States. Am J Sports Med.

[2] Kiapour AM, Murray MM (2014). Basic science of anterior cruciate ligament injury and repair. Bone Joint Res.

[3] Beynnon BD, Johnson RJ, Abate JA, Fleming BC, Nichols CE (2005). Treatment of anterior cruciate ligament injuries, part I. Am J Sports Med.

[4] Vaishya R, Agarwal AK, Ingole S, Vijay V (2015). Current trends in anterior cruciate ligament reconstruction: a review. Cureus.

[5] Prodromos CC, Joyce BT, Shi K, Keller BL (2005). A meta-analysis of stability after anterior cruciate ligament reconstruction as a function of hamstring versus patellar tendon graft and fixation type. Arthroscopy.

[6] Alswat MM, Khojah O, Alswat AM (2020). Returning to sport after anterior cruciate ligament reconstruction in physically active individuals. Cureus.

[7] Delay BS, Smolinski RJ, Wind WM, Bowman DS (2001). Current practices and opinions in ACL reconstruction and rehabilitation: results of a survey of the American Orthopaedic Society for Sports Medicine. Am J Knee Surg.

[8] Xie X, Liu X, Chen Z, Yu Y, Peng S, Li Q (2015). A meta-analysis of bone-patellar tendon-bone autograft versus four-strand hamstring tendon autograft for anterior cruciate ligament reconstruction. Knee.

[9] Goldblatt JP, Fitzsimmons SE, Balk E, Richmond JC (2005). Reconstruction of the anterior cruciate ligament: meta-analysis of patellar tendon versus hamstring tendon autograft. Arthroscopy.

[10] Li S, Su W, Zhao J, Xu Y, Bo Z, Ding X, Wei Q (2011). A meta-analysis of hamstring autografts versus bone-patellar tendon-bone autografts for reconstruction of the anterior cruciate ligament. Knee.

[11] Freedman KB, D'Amato MJ, Nedeff DD, Kaz A, Bach BR Jr (2003). Arthroscopic anterior cruciate ligament reconstruction: a metaanalysis comparing patellar tendon and hamstring tendon autografts. Am J Sports Med.

[12] Mohtadi NG, Chan DS, Dainty KN, Whelan DB (2011). Patellar tendon versus hamstring tendon autograft for anterior cruciate ligament rupture in adults. Cochrane Database Syst Rev.

[13] Mastrokalos DS, Springer J, Siebold R, Paessler HH (2005). Donor site morbidity and return to the preinjury activity level after anterior cruciate ligament reconstruction using ipsilateral and contralateral patellar tendon autograft: a retrospective, nonrandomized study. Am J Sports Med.

[14] Hardy A, Casabianca L, Andrieu K, Baverel L, Noailles T (2017). Complications following harvesting of patellar tendon or hamstring tendon grafts for anterior cruciate ligament reconstruction: systematic review of literature. Orthop Traumatol Surg Res.

[15] Schuette HB, Kraeutler MJ, Houck DA, McCarty EC (2017). Bone-patellar tendon-bone versus hamstring tendon autografts for primary anterior cruciate ligament reconstruction: a systematic review of overlapping meta-analyses. Orthop J Sports Med.

[16] Aglietti P, Giron F, Buzzi R, Biddau F, Sasso F (2004). Anterior cruciate ligament reconstruction: bone-patellar tendon-bone compared with double semitendinosus and gracilis tendon grafts. A prospective, randomized clinical trial. J Bone Joint Surg Am.

[17] Pinczewski LA, Lyman J, Salmon LJ, Russell VJ, Roe J, Linklater J (2007). A 10-year comparison of anterior cruciate ligament reconstructions with hamstring tendon and patellar tendon autograft: a controlled, prospective trial. Am J Sports Med.

[18] Thompson SM, Salmon LJ, Waller A, Linklater J, Roe JP, Pinczewski LA (2016). Twenty-year outcome of a longitudinal prospective evaluation of isolated endoscopic anterior cruciate ligament reconstruction with patellar tendon or hamstring autograft. Am J Sports Med.

[19] Lidén M, Ejerhed L, Sernert N, Laxdal G, Kartus J (2007). Patellar tendon or semitendinosus tendon autografts for anterior cruciate ligament reconstruction: a prospective, randomized study with a 7-Year follow-up. Am J Sports Med.

[20] Mohtadi NG, Chan DS (2019). A randomized clinical trial comparing patellar tendon, hamstring tendon, and double-bundle ACL reconstructions: patient-reported and clinical outcomes at 5-year follow-up. J Bone Joint Surg Am.

[21] Laxdal G, Kartus J, Hansson L, Heidvall M, Ejerhed L, Karlsson J (2005). A prospective randomized comparison of bone-patellar tendon-bone and hamstring grafts for anterior cruciate ligament reconstruction. Arthroscopy.

[22] Samuelsen BT, Webster KE, Johnson NR, Hewett TE, Krych AJ (2017). Hamstring autograft versus patellar tendon autograft for ACL reconstruction: is there a difference in graft failure rate? A meta-analysis of 47,613 patients. Clin Orthop Relat Res.

[23] Gabler CM, Jacobs CA, Howard JS, Mattacola CG, Johnson DL (2016). Comparison of graft failure rate between autografts placed via an anatomic anterior cruciate ligament reconstruction technique: a systematic review, meta-analysis, and meta-regression. Am J Sports Med.

[24] Huber R, Viecelli C, Bizzini M (2019). Knee extensor and flexor strength before and after anterior cruciate ligament reconstruction in a large sample of patients: influence of graft type. Phys Sportsmed.

[25] Aune AK, Holm I, Risberg MA, Jensen HK, Steen H (2001). Four-strand hamstring tendon autograft compared with patellar tendon-bone autograft for anterior cruciate ligament reconstruction. A randomized study with two-year follow-up. Am J Sports Med.

[26] Ageberg E, Roos HP, Silbernagel KG, Thomeé R, Roos EM (2009). Knee extension and flexion muscle power after anterior cruciate ligament reconstruction with patellar tendon graft or hamstring tendons graft: a cross-sectional comparison 3 years post surgery. Knee Surg Sports Traumatol Arthrosc.

[27] Webster KE, Feller JA, Hartnett N, Leigh WB, Richmond AK (2016). Comparison of patellar tendon and hamstring tendon anterior cruciate ligament reconstruction: a 15-year follow-up of a randomized controlled trial. Am J Sports Med.

[28] Leys T, Salmon L, Waller A, Linklater J, Pinczewski L (2012). Clinical results and risk factors for reinjury 15 years after anterior cruciate ligament reconstruction: a prospective study of hamstring and patellar tendon grafts. Am J Sports Med.

[29] Xergia SA, McClelland JA, Kvist J, Vasiliadis HS, Georgoulis AD (2011). The influence of graft choice on isokinetic muscle strength 4-24 months after anterior cruciate ligament reconstruction. Knee Surg Sports Traumatol Arthrosc.

[30] Ardern CL, Taylor NF, Feller JA, Webster KE (2014). Fifty-five per cent return to competitive sport following anterior cruciate ligament reconstruction surgery: an updated systematic review and meta-analysis including aspects of physical functioning and contextual factors. Br J Sports Med.

